# Women show enhanced proprioceptive target estimation through visual-proprioceptive conflict resolution

**DOI:** 10.3389/fpsyg.2024.1462934

**Published:** 2024-12-16

**Authors:** Anderson Barcelos de Melo, Jesus Landeira-Fernandez, Thomas Eichenberg Krahe

**Affiliations:** Departamento de Psicologia, Laboratório de Neurociência do Comportamento, Pontifícia Universidade Católica do Rio de Janeiro, Rio de Janeiro, Brazil

**Keywords:** multisensory integration, visuo-proprioceptive conflict, embodiment, mirror box illusion, mirror drawing, star-tracing, visuo-proprioceptive recalibration, visuomotor adaptation

## Abstract

To form a unified and coherent perception of the organism’s state and its relationship with the surrounding environment, the nervous system combines information from various sensory modalities through multisensory integration processes. Occasionally, data from two or more sensory channels may provide conflicting information. This is particularly evident in experiments using the mirror-guided drawing task and the mirror-box illusion, where there is conflict between positional estimates guided by vision and proprioception. This study combined two experimental protocols (the mirror-box and the mirror-guided drawing tasks) to examine whether the learned resolution of visuo-proprioceptive conflicts in the mirror-guided drawing task would improve proprioceptive target estimation of men and women during the mirror-box test. Our results confirm previous findings of visual reaching bias produced by the mirror-box illusion and show that this effect is progressively reduced by improvement in the mirror drawing task performance. However, this was only observed in women. We discuss these findings in the context of possible gender differences in multisensory integration processes as well as in embodiment.

## 1 Introduction

The concept of embodiment refers to the idea that cognitive processes are deeply rooted in the body’s interactions with the environment, and that perception is intrinsically linked to both sensory and motor processes (Fuchs and Schlimme, 2009; Craighero, 2014; Varela et al., 2016; Shapiro, 2019). From a more specific perspective, embodiment represents the subjective sensation associated with possessing and disposing of one’s own body (Longo et al., 2008; Medina et al., 2015). In this sense, several authors have shown the importance of research on embodiment for understanding the mechanisms of multisensory integration (Botvinick and Cohen, 1998; Holmes et al., 2004; Holmes and Spence, 2005; Miall and Cole, 2007; Longo et al., 2008; Otsuru et al., 2014; Diers et al., 2015; Medina et al., 2015; Vecchiato et al., 2015; Liu and Medina, 2017, 2018, 2021; Carey et al., 2019; Giroux et al., 2019; Leach and Medina, 2022; Ambron and Medina, 2023; Ding et al., 2023). Additionally, findings show the relevance of the sense of embodiment in various clinical applications, such as the treatment of “phantom pain” in amputees (Ramachandran et al., 1995; Schmalzl et al., 2013; Collins et al., 2018; Kundi and Spence, 2023), improvement of motor function after stroke (Altschuler et al., 1999; Thieme et al., 2018; Lee and Lee, 2019; Kundi and Spence, 2023), management of body image and eating disorders (Griffen et al., 2018), alleviation of motor symptoms associated with multiple sclerosis (Tekeoglu Tosun et al., 2021), and rehabilitation of complex regional pain syndrome (Al Sayegb et al., 2013).

The mirror therapy technique stands out as a powerful tool for understanding the processes related to multisensory integration and embodiment, particularly to those, but not limited to, the alleviation of symptoms associated with “phantom limb” pain (Ramachandran et al., 1995; Ramachandran and Rodgers-Ramachandran, 1996; Dohle et al., 2019). Amputee patients undergoing this therapy often report that the sight of the reflection of their remaining limb in motion or being stimulated is perceived as their missing limb - a phenomenon termed mirror visual feedback (Ramachandran et al., 1995; Ramachandran and Rodgers-Ramachandran, 1996; Deconinck et al., 2015). Despite the established effectiveness of the technique in clinical settings, the underlying mechanisms still need further elucidation (Dohle et al., 2019). Nevertheless, it is widely acknowledged that the process of embodiment during mirror feedback involves the integration of various sensory modalities such as kinesthesia (movement), touch, vision, and proprioception (Holmes et al., 2004). The latter encompasses sensory information from the joints, muscles, and tendons, contributing to our perception of body positioning and movement in space. It is important to note that, in the literature, the terms proprioception and kinesthesia are sometimes used interchangeably (Stillman, 2002), whereas for others, kinesthesia specifically denotes the perception of movement (Swanik et al., 2004; Swanik et al., 2002). There is also an understanding that kinesthesia is a subset of proprioception (Lephart et al., 1997; Myers et al., 1999). Yet, most commonly, proprioception is defined broadly, to include the sense of movement (Han et al., 2016; Tuthill and Azim, 2018; Blum et al., 2021; Moon et al., 2021), playing a pivotal role in the embodiment process during mirror therapy (Holmes et al., 2004; Holmes and Spence, 2005; Medina et al., 2015; Leach and Medina, 2022). Beyond its clinical applications, the concept underpinning mirror therapy serves as a valuable research tool for investigating visual-proprioceptive conflicts and expand our understanding of multisensory processes and embodiment in healthy individuals (Holmes et al., 2004; Holmes and Spence, 2005; Medina et al., 2015; Liu and Medina, 2021; Leach and Medina, 2022). For instance, discordant visual and proprioceptive-placement information significantly impacts the accuracy of target-reaching movements made with the unseen arm in non-clinical samples of young adults (visual capture; Holmes and Spence, 2005; Holmes et al., 2004).

The mirror drawing task stands as another common method to induce visuo-proprioceptive conflicts, wherein vision supersedes proprioception in resolving such conflicts (Lajoie et al., 1992; Balslev et al., 2004; Miall and Cole, 2007; Miall et al., 2021). A good illustration of this phenomenon is observed in the star-tracing drawing task where individuals must outline the reflected image of a six-pointed star, allowing for the assessment of how visual and proprioceptive information are integrated and modified through learning processes (Lajoie et al., 1992). Intriguingly, research has demonstrated that this ability is compromised in individuals afflicted with mirror agnosia and mirror ataxia (Ramachandran et al., 1997; Binkofski et al., 1999), whereas patients experiencing selective loss of proprioceptive afferent inputs show little to no visuo-proprioceptive conflicts (Lajoie et al., 1992; Balslev et al., 2004; Miall and Cole, 2007; Miall et al., 2021). In contrast, healthy individuals fully experience this conflict and must learn the new skill through visuomotor adaptation. This process occurs dynamically with the internal map recalibration, which iteratively resolves the visuo-proprioceptive conflict (Lajoie et al., 1992; Guedon et al., 1998; Balslev et al., 2004; Cressman and Henriques, 2009; Henriques and Cressman, 2012; Ruttle et al., 2016).

Visuomotor adaptation occurs when an individual is required to adjust their motor output in response to a mismatch between visual feedback and actual motor performance. This conflict is typically introduced experimentally by altering the visual representation of movement (Shadmehr and Mussa-Ivaldi, 1994; Redding and Wallace, 2006; Cressman and Henriques, 2009; Krakauer, 2009). As before mentioned, this kind of adaptation has been shown to occur in the mirror drawing task, where the participants are asked to trace a geometrical shape through its image in the mirror while the direct vision of the drawing hand is blocked (Lajoie et al., 1992; Milner, 1998; Balslev et al., 2004; Miall et al., 2021). After training, the brain adjusts the proprioceptive map of the body to align more closely with the altered visual feedback. This recalibration is thought to involve both short-term and long-term adjustments in sensory integration and neural plasticity, allowing the individual to perform the task more accurately as the brain recalibrates its sensory systems (Shadmehr and Krakauer, 2008; Cressman and Henriques, 2010; Ostry et al., 2010; Ruttle et al., 2016).

Research has demonstrated that men and women differ in visuomotor adaptation and in their ability to resolve visuo-proprioceptive conflicts. It is well established that men typically perform better in visuospatial and visuomotor tasks, and that sex differences also extend to the perception of visual illusions and body representation (Linn and Petersen, 1985; Viaud-Delmon et al., 1998; Peled et al., 2000; Barnett-Cowan et al., 2010; Cadieux et al., 2010; Egsgaard et al., 2011; Thakkar et al., 2011; Eshkevari et al., 2012; Ferri et al., 2014; Longo, 2022; Fioriti et al., 2024). Additionally, men and women differ in the prevalence of psychopathological conditions such as bulimia and anorexia nervosa, with women exhibiting up to 15 times the prevalence of anorexia compared to men (Qian et al., 2022). These disorders are strongly associated with multisensory integration deficits and body representation distortions, a phenomenon that also occurs in healthy individuals when exposed to discordant visual and proprioceptive information (Longo, 2022, 2023; Malighetti et al., 2022; Brizzi et al., 2023; Fusco et al., 2023; Navas-León et al., 2023). Thus, investigating visuo-proprioceptive conflicts through the lens of sex differences may offer valuable insights into the perceptual mechanisms underlying body representation distortions.

To further shed light on the specific roles of vision and proprioception in how the visuo-proprioceptive conflicts are resolved and given that previous findings show that visuomotor adaptation is followed by proprioceptive recalibration (Cressman and Henriques, 2009, 2010, 2011; Cressman et al., 2010; Henriques and Cressman, 2012; Flannigan et al., 2018), we integrated both the mirror box and the six-pointed star approaches to assess the visuo-proprioceptive recalibration in a sample of non-clinical participants. Furthermore, given previous findings suggesting sex differences in the perception of embodiment, visual illusions and visuomotor tasks (Linn and Petersen, 1985; Viaud-Delmon et al., 1998; Barnett-Cowan et al., 2010; Cadieux et al., 2010; Egsgaard et al., 2011), we also compared the performance of men and women.

## 2 Methods

### 2.1 Participants

Fifteen subjects (7 women and 8 men, mean age 42.3 ± 3.69 years) were recruited to participate in the study. All participants were right-handed, with no visual problems or with corrected vision and naïve to the purpose of the study. The study was approved by the Brazilian Ethics Committee (CEP/CONEP, # 63845022.3.0000.5281), ensuring that all procedures complied with ethical guidelines. All participants gave their informed consent prior the start of data collection.

### 2.2 Materials

A mirror box like the one used by Holmes et al. (2004) and Holmes and Spence (2005) was used for a task in which the participant should reach toward a target position with its hidden hand (reaching movement task; Holmes et al., 2004; Holmes and Spence, 2005). Briefly, a wooden parallelepiped (45 width × 45 length × 20 height, cm) without two opposite sidewalls was placed on a table (Supplementary Figure S1). The outward face of one of the remaining sidewalls had a slot to accommodate a removable mirror (45 cm × 30 cm). Three marks, only visible to the experimenter, were placed 5, 12, and 26 cm to the left of the mirror. The middle mark (12 cm) was considered the target position and the other two starting positions. To indicate the location of the target position to the participant, a cardboard with a downward arrow was positioned on the top surface of the parallelepiped (Supplementary Figure S1). A black cloth was used to cover the participant’s right arm and shoulder, occluding the view of their right hand.

For the star-tracing task, a second wooden parallelepiped (45 width × 30 length × 20 height, cm), without two opposite sidewalls (30 length × 20 height, cm), and with a rectangular opening on one of the remaining sidewalls, was placed 5 cm to the right of the mirror (Supplementary Figure S2). A tablet (Samsung Galaxy Tab S6 Lite) was positioned inside the parallelepiped allowing the participant to see its reflection while preventing from looking directly at it (Supplementary Figure S2). An image of a six-pointed star (2,490 × 3,510 pixels, 300 dpi) was displayed on the tablet for the star tracing task (Supplementary Figures S2, S3).

### 2.3 Procedures

**Figure 1:**
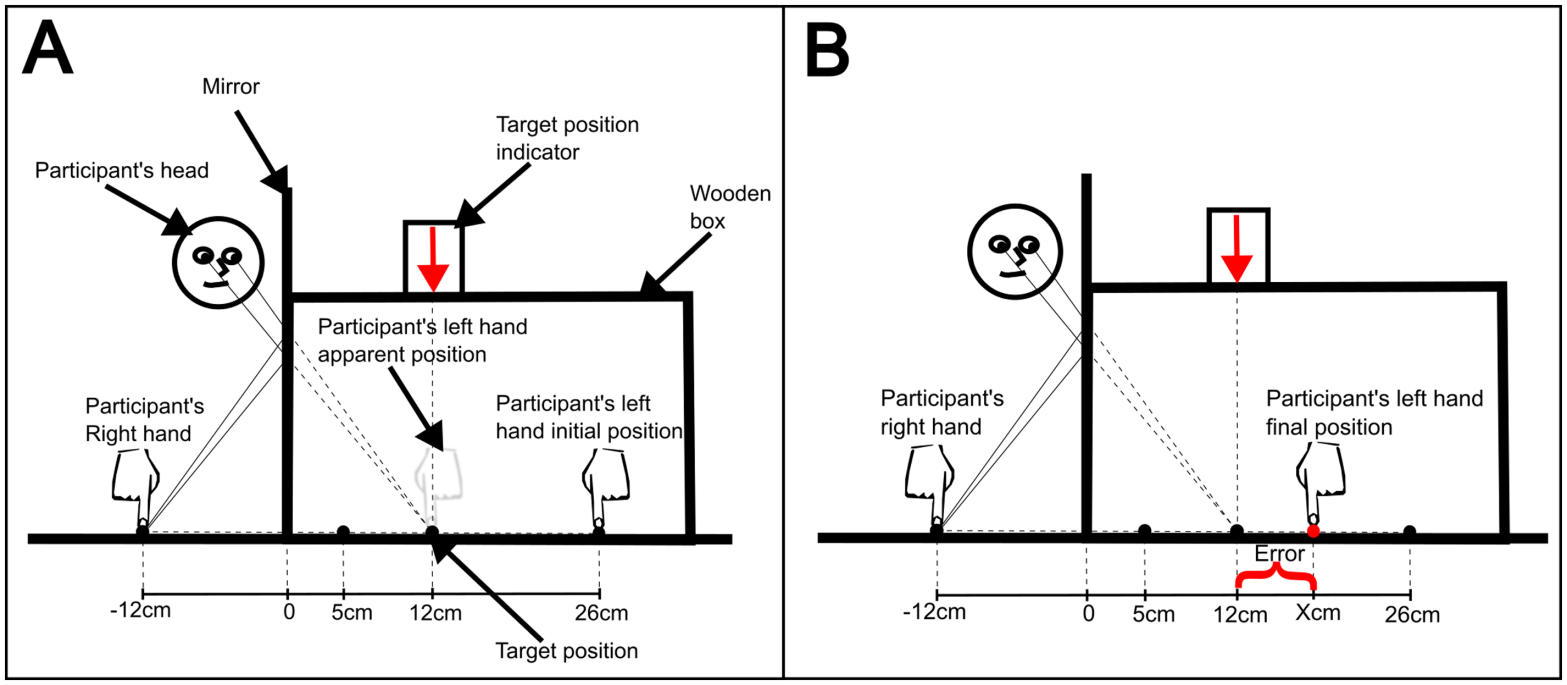
Schematic of target-reaching task, from the researcher’s perspective. **(A)** Initial task setup at the initial position of 26 cm. **(B)** Measurement of reaching error, after the participant’s reaching attempt.

For the reaching movement task, the participant sat at the table facing the mirror box and was asked to put their left arm inside the mirror box and the index finger of the other arm 12 cm to the right of the mirror (Figure 1). The experimenter covered the participant’s left arm and shoulder with the black cloth and sat on the other side of the table facing the participant. Next, the experimenter placed the participant’s left index finger (the one inside the box) at either 5 or 26 cm starting positions. At this first stage, the mirror was not positioned in the slot (no-mirror condition) and the participant was instructed to tap both index fingers synchronously at a frequency of 170 BPM (2.83 Hz) defined by a metronome (Medina et al., 2015). After 6 s, the experimenter asked the participant to reach, with his or her left index finger, the target position inside the box (12 cm), which was indicated by the downward arrow sign on top of the box. Then the experimenter measured the distance (cm) from the target to the participant’s reached position (error). The task was repeated until 5 measurements were completed for each start position (5 and 26 cm) alternately, totaling 10 attempts. The same procedure and number of repetitions were done with the mirror inserted in the slot. While synchronously tapping the participant was instructed to keep looking at the reflection of their right hand on the mirror, which induced the mirror box illusion (mirror condition; Figure 1). On the first day, there was a training session to familiarize the participants with the task (Holmes and Spence, 2005). This was a short session, no longer than 5 min, which comprised the reaching task in the ‘no mirror’ condition and an exposure to the mirror-box illusion. Such session was useful to correct the way the participants executed the reaching movements. They were instructed to make an intuitively single and continuous movement, with no pauses.

**Figure 2:**
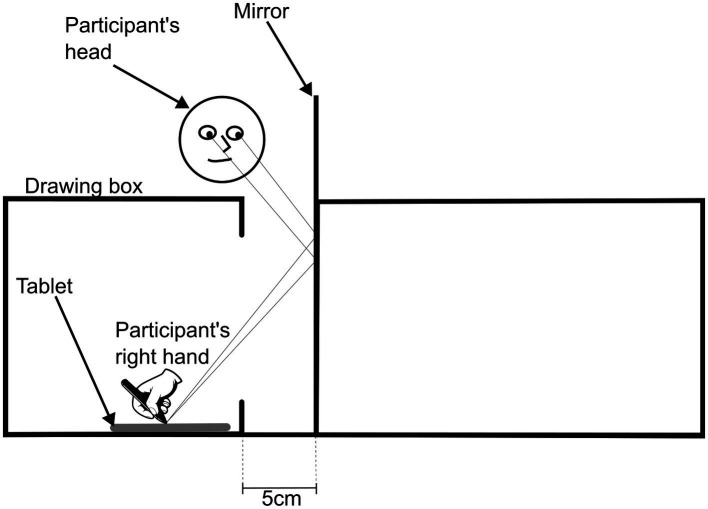
Schematic of the star-tracing task, from the researcher’s perspective.

Next, with the star-tracing setup in place, the participant was asked to draw the outline of the 6-pointed star looking at the reflection of the image (Figure 2). The experimenter positioned the tip of the pen at the top point of the star template and the participant had to choose whether they would like to outline the star in a counter-or clockwise direction, maintaining the same orientation in subsequent drawings. They were asked to complete the drawing the best way possible and in the shortest period. The participant was asked to repeat this sequence 10 times and, for each one, the image was captured, and the completion time recorded. Prior to the star-tracing task, each participant had to do the outlining of the star template three times with direct vision to the tablet.

After completing the star-tracing drawings, the participant repeated the protocol for the reaching target task in the ‘mirror’ condition and, right after, the same protocol, but in the ‘no-mirror’ condition (Supplementary Figure S4). The same sequence of experiments was repeated two more times, with a minimum interval of 24 h and a maximum of 72 h between sessions (36.8 ± 3.2 h).

### 2.4 Data analysis

Data analysis was performed using Python (version 3.10.10) and Jamovi software (version 2.2.5). Reaching task results were analyzed using repeated-measures ANOVA (rANOVA). The analysis included ‘sex’ (men and women) as the between-subject factor, while ‘mirror’ (mirror and no-mirror), ‘initial position’ (left hand at 5 or 26 cm), ‘reaching phase’ (before or after star-tracing task within a given day), and ‘session’ (first, second or third day of testing) were the within-subjects factor. Data from the star-tracing task were analyzed using a Python script (Supplementary materials). An accuracy index (I_acc_) was calculated based on the number of pixels drawn within and outside the star’s template, as well as the total number of pixels.


$$I_{acc}=\frac{p_{\mathrm{ideal}}}{p_{\mathrm{drawn}}}\times \frac{p_{\mathrm{within}}}{p_{\mathrm{draw}}}\text{,}$$

where:

-
$p_{\mathrm{drawn}}$ is the total number of pixels drawn (within and outside the star outline).
-
$p_{\mathrm{within}}$ is the number of pixels drawn within the star outline limits.
-
$p_{\mathrm{ideal}}$ is the total number of pixels of a perfect outline (12 straight lines).

A second index was calculated to assess the speed of execution for the star-tracing task. This was done by comparing tracing times with participants looking to the mirror against those obtained with direct vision of the star template (i.e., not its reflection).


$$I_{\mathrm{speed}}=\frac{\Delta t_{\mathrm{base}}}{\Delta t_{\mathrm{drawing}}}\text{,}$$

where:

-
$\Delta t_{\mathrm{base}}$ is the mean time (3 trials) to outline the star looking at it.
-
$\Delta t_{\mathrm{drawing}}$ is the time to outline the star’s reflection.

Indices of 0.0 indicate the lowest accuracy and speed values, while indices of 1.0 represent the highest accuracy and speed. To evaluate participants’ performance in the star-tracing task, a repeated-measures ANOVA (rANOVA) was conducted, with ‘sex’ as the between-subjects factor and ‘session’ (first, second or third day of testing) as the within-subject factor. The effect size for both rANOVA factors was calculated using partial eta squared (ηp^2^). *Post hoc* pairwise comparisons were adjusted for multiple comparisons using the Bonferroni-Holm method. Additionally, a correlation analysis was performed between the indices and errors made in the reaching movement task. All data are presented as mean ± SEM, unless otherwise specified. Statistical significance was set at *p* < 0.05.

## 3 Results

**Figure 3:**
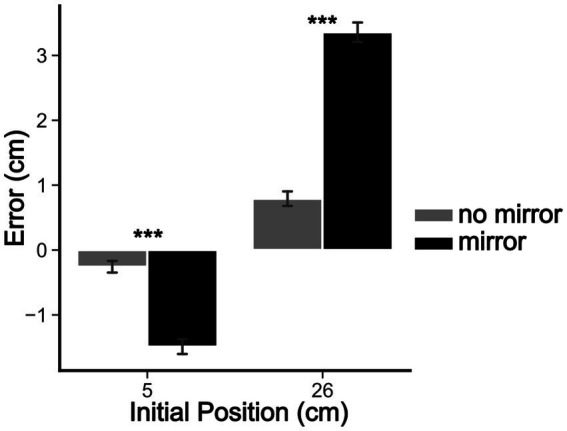
Visual capture effect. Bar graph depicting the mean target estimation errors made by participants in the reaching task for the mirror and no-mirror conditions. The *x*-axis shows the initial position of the hidden left hand in centimeters, and the *y*-axis shows the mean reaching errors in cm. Bars are means ± SEM. *p < 0.05; ***p < 0.001.

**Figure 4:**
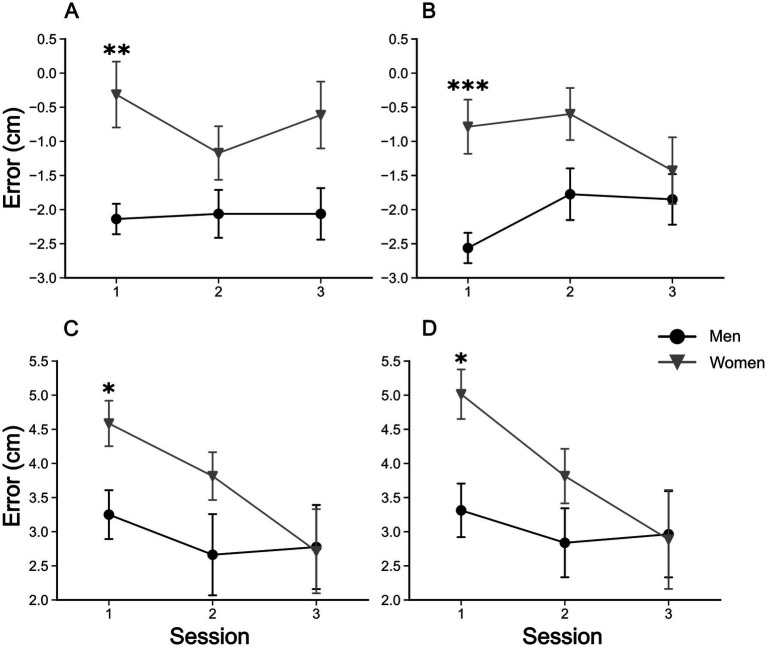
Evolution of reaching errors in the ‘mirror’ condition. Upper and lower panels show the errors for ‘initial position’ of 5 and 26 cm, respectively. Left and right panels show the errors for ‘reaching phase’ before and after the mirror drawing task, respectively. **(A)** Mean reaching errors for ‘reaching phase’ before the mirror drawing task, at the ‘initial position’ 5 cm. **(B)** Mean reaching errors for ‘reaching phase’ after the mirror drawing task, at the ‘initial position’ 5 cm. **(C)** Mean reaching errors for ‘reaching phase’ before the mirror drawing task, at the ‘initial position’ 26 cm. **(D)** Mean reaching errors for ‘reaching phase’ after the mirror drawing task, at the ‘initial position’ 26 cm. Symbols are means ± SEM. *p < 0.05, **p < 0.01, and ***p < 0.001.

The five-way rANOVA for the reaching task showed a significant interaction among sex, mirror, initial position, reaching phase, and session [*F*(2, 146) = 4.079, *p* < 0.02, ηp^2^ = 0.053]. No significant four-way interactions were detected. We found significant three-way interactions among ‘sex’ × ‘mirror’ × ‘reaching phase’ [*F*(1, 73) = 4.777, *p* < 0.05, ηp^2^ = 0.061], ‘mirror’ × ‘initial position’ × ‘reaching phase’ [*F*(1, 73) = 4.989, *p* < 0.05, ηp^2^ = 0.064], and ‘initial position’ × ‘reaching phase’ × ‘session’ [*F*(2, 146) = 4.097, *p* < 0.05, ηp^2^ = 0.053]. Significant two-way interactions were also found among ‘sex’ × ‘mirror’ [*F*(1, 73) = 6.846, *p* < 0.05, ηp^2^ = 0.086], ‘sex’ × ‘session’ [*F*(2, 146) = 3.796, *p* < 0.05, ηp^2^ = 0.049], and ‘initial position’ × ‘session’ [*F*(2, 146) = 11.30038, *p* < 0.001, ηp^2^ = 0.134]. Finally, the main effects of ‘sex’ [*F*(1, 73) = 4.09, *p* < 0.05, ηp^2^ = 0.053], ‘mirror’ [*F*(1, 73) = 31.04, *p* < 0.001, ηp^2^ = 0.298], ‘initial position’ [*F*(1, 73) = 174.70, *p* < 0.001, ηp^2^ = 0.705], and ‘session’ [*F*(2,146) = 8.57, *p* < 0.001, ηp^2^ = 0.105] were detected. No other significant effects were found (all *p*-values >0.05). *Post hoc* comparisons revealed that the visual capture effect (Figure 3) occurred in all sessions of experiments, with all comparisons reaching *p* < 0.001 corrected by the Bonferroni-Hom method. Additionally, in the ‘mirror’ condition (with mirror), there were significant differences of reaching errors between men and women in the first session (1st day of testing), with women committing larger errors than man for the 26 cm ‘initial position’ (before mirror drawing: Mean Difference = 1.3 cm, *p*_Holm_ < 0.05; after: Mean Difference = 1.7 cm, *p*_Holm_ < 0.05). This significant difference disappeared in the next sessions since a significant decrease in reaching errors was found for women (26 cm ‘initial position’) from the first to third session (before mirror drawing: Mean Difference = 1.9 cm, *p*_Holm_ < 0.05; after mirror drawing: Mean Difference = 2.1 cm, *p*_Holm_ < 0.05). These results can be better visualized in Figure 4.

**Figure 5:**
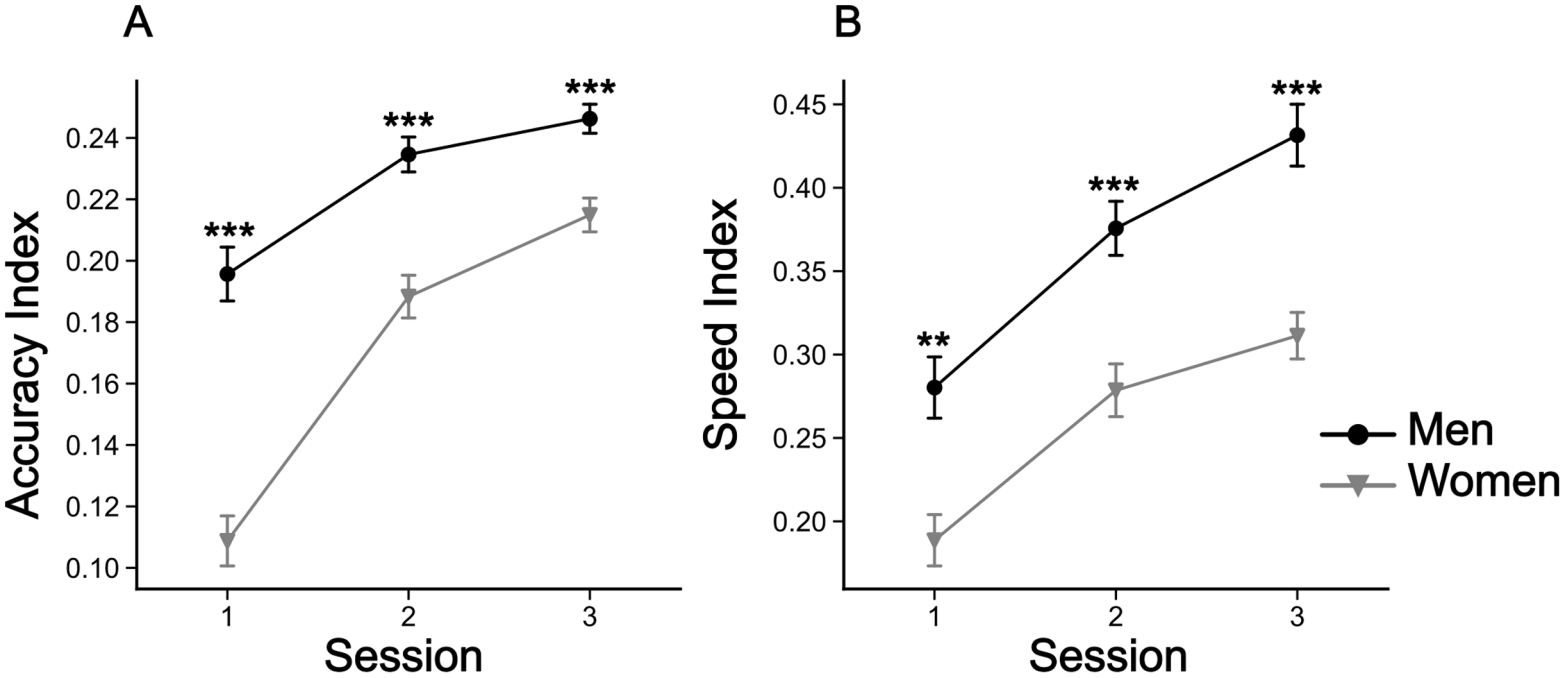
Evolution of accuracy and speed indices for the mirror drawing task. **(A)** Mean accuracy indices for men and women in the mirror drawing task across sessions. **(B)** Mean speed indices for men and women in the mirror drawing task across sessions. Symbols are means ± SEM. **p < 0.01, and ***p < 0.001.

The two-way rANOVA for the accuracy index revealed a significant the interaction for ‘sex’ × ‘session’ [*F*(2,296) = 24.2, *p* < 0.001, ηp^2^ = 0.141], as well as for the main effects of ‘sex’ [*F*(1,148) = 43.2, *p* < 0.001, ηp^2^ = 0.226], and ‘session’ [*F*(2,296) = 195.6, *p* < 0.001, ηp^2^ = 0.569]. For the speed index we found no significant interaction between ‘sex’ and ‘session. However, significant effects were found for ‘sex’ [*F*(1,148) = 23.1, *p* < 0.001, ηp^2^ = 0.135] and ‘session’ [*F*(2,296) = 132.61, *p* < 0.001, ηp^2^ = 0.473]. Although both sexes showed a clear improvement in both accuracy and speed in the mirror drawing task over testing sessions, the overall performance of women was always worse compared to that observed for men (Figures 5A,B). For representative man and woman mirror drawing performances, please see Supplementary Figure S5. Moreover, women showed the largest improvement in mirror drawing accuracy (Figure 5).

**Figure 6:**
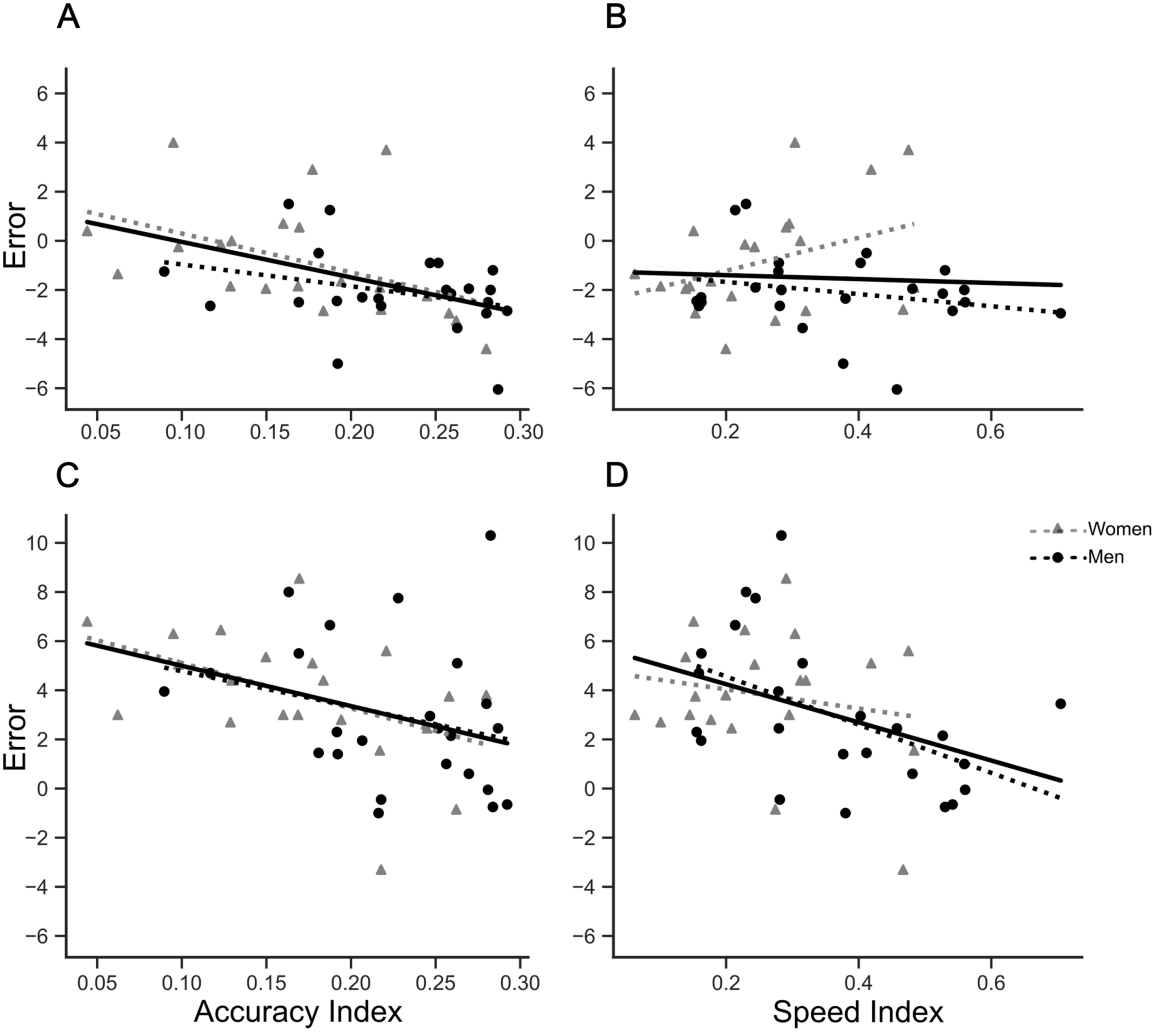
Correlations between reaching errors in ‘mirror’ condition and mirror drawing indices. Upper and lower panels show scatter plots and tendency lines for ‘initial position’ of 5 and 26 cm, respectively. Left and right panels show the scatter plot and tendency lines for mirror drawing accuracy and speed indices, respectively. **(A)** Correlation between reaching errors at 5 cm ‘initial position’ and Accuracy Indices. Women: *r* = −0.456, *p* < 0.05; Men: No significant correlation. **(B)** Correlation between reaching errors at 5 cm ‘initial position’ and Speed Indices. No significant correlations found for women and men. **(C)** Correlation between reaching errors at 26 cm ‘initial position’ and Accuracy Indices. Women: *r* = −0.462, *p* < 0.05; Men: No significant correlation. **(D)** Correlation between reaching errors at 26 cm ‘initial position’ and Speed Indices. Women: No significant correlation. Men: *r* = −0.504, *p* < 0.05. Solid and traced lines represent linear fits. Accuracy and speed indices showed a positive correlation of 0.579 (*p* < 0.001).

Having found significant differences in performance over sessions and between sexes for both reaching target and mirror drawing tasks, we next investigated possible relationships between reaching errors and mirror drawing indices. Since no significant effect for ‘reaching phase’ [*F*(1,73) = 0.578, *p* = 0.449, ηp^2^ = 0.008], nor interaction between ‘reaching phase’ × ‘session’ [*F*(2,146) = 0.361, *p* = 0.697, ηp^2^ = 0.005] was found, reaching target errors before and after mirror drawing within each session were aggregated as average errors. Due to the aim of the study and space limitations, only correlations for the mirror condition are showed (Figure 6). Interestingly, correlations involving accuracy indices (Figures 6A,C) were found to be significant for women (5 cm, *r* = −0.456, *p* < 0.05; 26 cm = −0.462, *p* < 0.05), but not for men (5 cm, *r* = −0.305, *p* = 0.15; 26 cm, *r* = −0.264, *p* = 0.21). In contrast, we have found no statistical significance for correlations between speed index values and reaching errors (Figures 6B,D), except for men in the farthest reaching errors (Figure 6D, men, *r* = −0.504, *p* < 0.05; women, *r* = −0.184, *p* = 0.43).

## 4 Discussion

Our results support previous studies showing that the mirror box illusion alters participants’ perception of the hidden hand’s position. Given that participants consistently committed greater mean reaching errors in the ‘mirror’ condition, and that such errors were systematically made through smaller reaching movements, we can state that the perceived hidden hand’s initial position was shifted toward the visually informed location, in accordance with the visual capture phenomenon reported by other researchers (Holmes et al., 2004; Holmes and Spence, 2005; Medina et al., 2015; Liu and Medina, 2018). This effect was evident in both men and women during the mirror-box illusion, supporting the notion that the brain integrates visual and proprioceptive information in a statistical way and that the visual capture results from this integrative process in presence of proprioceptive drift. Since the precision (reliability) of proprioceptive inputs are diminished because of its greater variance, which in turn is induced by the blocked vision of the performing hand, vision’s relative weight is increased, creating the visual bias (Wann and Ibrahim, 1992; Van Beers et al., 1999; Ernst and Banks, 2002; Brown et al., 2003; Ernst and Bülthoff, 2004; Holmes and Spence, 2005; Guerraz et al., 2012). The novelty of our findings is showing that this embodiment effect was progressively mitigated in women by the introduction of the star-tracing task. In line with previous research on visuomotor adaptation (Cressman and Henriques, 2009, 2015; Cressman et al., 2010; Salomonczyk et al., 2011; Henriques and Cressman, 2012; Ruttle et al., 2016; Flannigan et al., 2018; Bouchard and Cressman, 2021; Wijeyaratnam et al., 2022), our results indicate that training-induced recalibration can affect subsequent tasks, supporting the idea that recalibration is not task-specific but can be applied to other contexts involving similar sensory conflicts. Yet, this was only true for women and if we consider errors associated to the furthest distance from the target. One limitation of our study was to not include a control group, that would not be a participant in the star-tracing task. However, this issue was overcome by the comparison between the reaching errors in the ‘mirror’ and ‘no mirror’ conditions. If the improvement in the reaching errors was to be attributed to the participants learning how to better perform in the reaching task, we should have observed a significant reduction in the ‘no mirror’ condition as well, which was not true.

Visuo-proprioceptive conflicts impacting the accuracy of target-reaching movements made with the unseen hand (Holmes et al., 2004; Holmes and Spence, 2005; Medina et al., 2015; Liu and Medina, 2018) can also be influenced by one’s embodiment experience (Medina et al., 2015; Liu and Medina, 2017, 2018). Another limitation of our study is that we did not investigate embodiment measures associated with the subjective experience of the mirror-box illusion and how it is related to visuo-proprioceptive recalibration. We would expect that embodiment measures should decrease after the visuo-proprioceptive recalibration, which would reinforce the association between multisensory integration processes and body ownership distortions.

Lajoie et al. (1992) showed in their seminal paper that visual-proprioceptive conflicts occur in the mirror star-tracing task. It was demonstrated that a patient with severe proprioceptive deficits outperformed controls from the outset, while controls only achieved similar performances after resolving visuo-proprioceptive conflicts through mirror star-tracing practice. This suggests the necessity of implicit motor learning to recalibrate their visual-proprioceptive spatial representation. This understanding is supported by another study in which TMS was used to disrupt the proprioceptive processing of participants, thereby improving their performance in mirror-guided tasks (Balslev et al., 2004). Moreover, it was later demonstrated that at the beginning of mirror-guided tasks, where visual-proprioceptive conflicts are stronger, there is significant suppression of primary somatosensory cortex activation by prefrontal cortex inputs, which wanes with task repetition (Bernier et al., 2009).

A key aspect of our study was the focus on sex differences in visuo-proprioceptive integration. As outlined in the introduction, previous research has shown that men and women differ in their ability to resolve sensory conflicts, particularly in tasks that involve visuospatial and visuomotor skills (Linn and Petersen, 1985; Viaud-Delmon et al., 1998; Barnett-Cowan et al., 2010; Cadieux et al., 2010; Egsgaard et al., 2011; Fioriti et al., 2024). In alignment with these results, we show that women experienced higher levels of visuo-proprioceptive conflict at first, but also exhibited greater adaptation after training on the star-tracing task. This is confirmed by the significant reduction in the reaching errors and the greater improvement of women found in the star-tracing. In this task they improved the initial mean accuracy index (Iacc) by 90%, in contrast with an increase of 20% for men, while the speed index showed a similar improvement for both sexes (50% for women and 54% for men). Finally, reinforcing this finding, the correlation analysis showed that the reaching errors and the accuracy index are significantly and negatively correlated only for women, adding up to the evidence for a visuomotor adaptation followed by a decrease in the visuo-proprioceptive conflict experienced by women.

The observed sex differences in visuo-proprioceptive conflicts and recalibration also have implications for understanding perceptual distortions in body representation, particularly in the context of eating disorders. As stated earlier, women are disproportionately affected by disorders such as bulimia and anorexia nervosa, which are associated with multisensory integration deficits and distorted body representation (Eshkevari et al., 2012; Malighetti et al., 2020; Longo, 2022; Qian et al., 2022). Our findings suggest that women are more susceptible to visual-proprioceptive conflicts, but that they can adapt to match the levels experienced by men. Research on interventions for eating disorders and its related body representation distortion, such as mirror therapy and virtual reality therapies (Griffen et al., 2018; Malighetti et al., 2020; Matamala-Gomez et al., 2021; Riva et al., 2021; Navas-León et al., 2024), suggest that the greater magnitude of visuo-proprioceptive recalibration observed in women may also have therapeutic implications, as interventions aimed at improving multisensory integration could be tailored to leverage this result.

By using a protocol that combines mirror-box reaching target (Holmes et al., 2004; Holmes and Spence, 2005) and mirror star-tracing tasks (mirror drawing; Lajoie et al., 1992), we demonstrate that resolving visual-proprioceptive conflicts in the latter improves women’s performance in reaching target estimations. However, it is important to note that the improvement in performance in reaching the target from the farthest position is noticeable only across the three sessions of the experiment, not within the same day. This suggests that recalibration of visual-proprioceptive target estimation depends on the performance improvement observed in the mirror star-tracing task over time, indicating a cognitive process involving long-term consolidation of implicit memory (Salomonczyk et al., 2011; Tibi et al., 2013; Voges et al., 2015; Maksimovic and Cressman, 2018; Neville and Cressman, 2018; Hadjiosif et al., 2023). Previous studies demonstrating improvement in mirror star-tracing task performance have shown that this change relies on implicit memory consolidation (Corkin, 1968, 2002; Shadmehr et al., 1998). Furthermore, the observation that this improvement in performance in reaching target estimation was evident only in women also calls for further investigation. While embodiment differences between men and women have been previously demonstrated (Egsgaard et al., 2011), the understanding of the underlying mechanisms remains elusive. Nevertheless, we cannot dismiss the potential clinical applications of visual-proprioceptive conflict mitigation, especially in psychiatric conditions such as eating disorders, where heightened embodiment and multisensory integration deficits are well documented (Eshkevari et al., 2012; Keizer et al., 2014; Zopf et al., 2016; Serino and Dakanalis, 2017; Malighetti et al., 2020; Matamala-Gomez et al., 2021; Riva et al., 2021; Navas-León et al., 2024).

In conclusion, this study provides novel insights into how visuo-proprioceptive recalibration generalizes across tasks and highlights the importance of considering sex differences in multisensory integration. Our results suggest that women may experience greater visuo-proprioceptive conflicts, but also that they can adapt to match men’s levels, which has potential implications not only for understanding perceptual distortions in healthy participants, but also for clinical populations, such as those with eating disorders. By investigating the mechanisms underlying these differences, we can develop more targeted interventions for individuals with multisensory integration deficits.

## Data Availability

The original contributions presented in the study are included in the article/Supplementary material, further inquiries can be directed to the corresponding author.
