# Supplementary figures and images for: Women show enhanced proprioceptive target estimation through visual-proprioceptive conflict resolution
Source: Front Psychol. 2024 Dec 16;15:1462934. doi: 10.3389/fpsyg.2024.1462934 (PMC11684459; doi:10.3389/fpsyg.2024.1462934)

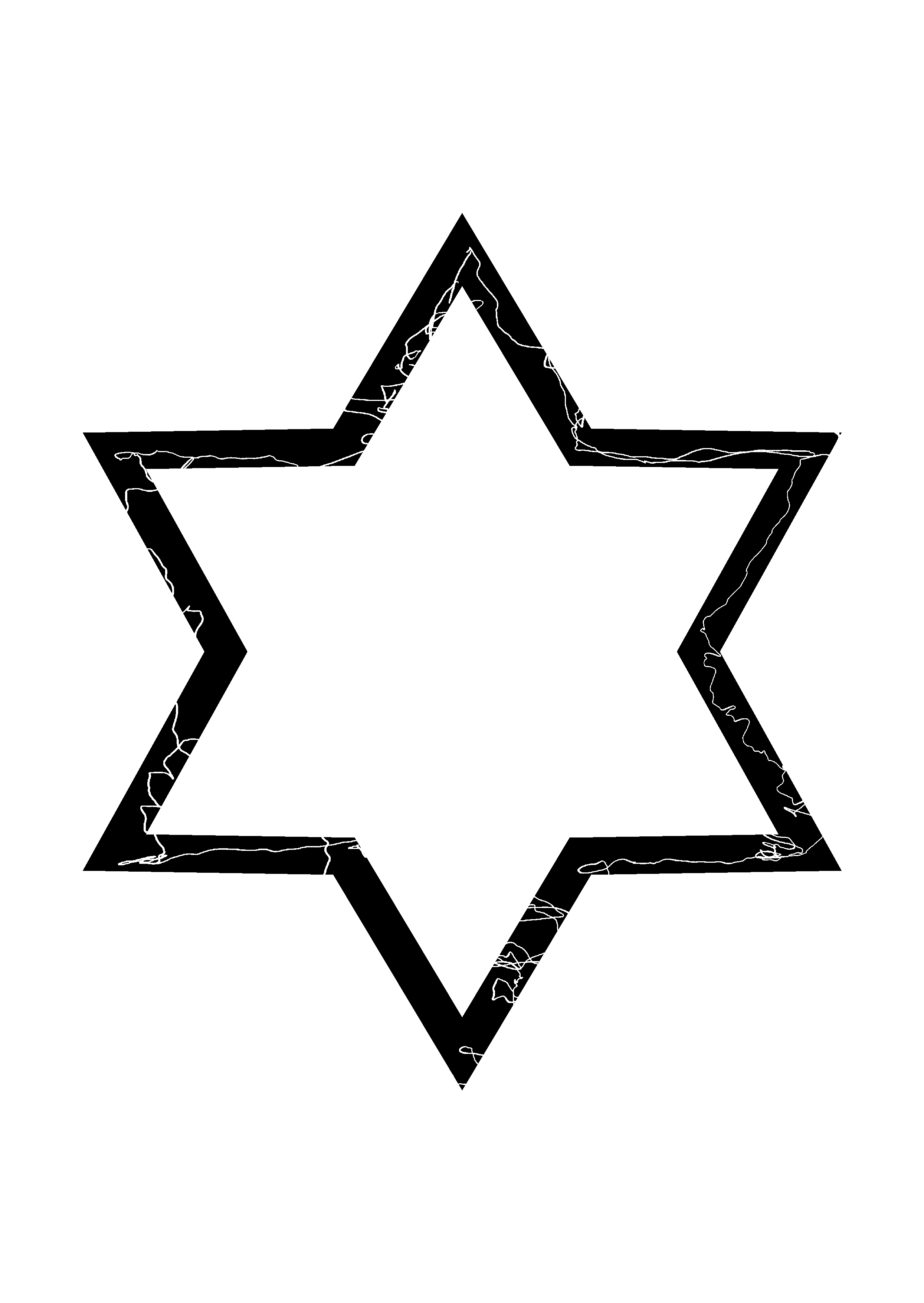

Supplement: Supplementary file 1 [file Data_Sheet_1.ZIP › AnalyzeStarDraw_PythonScript/AnalyzedImages/example_draw_in.png]

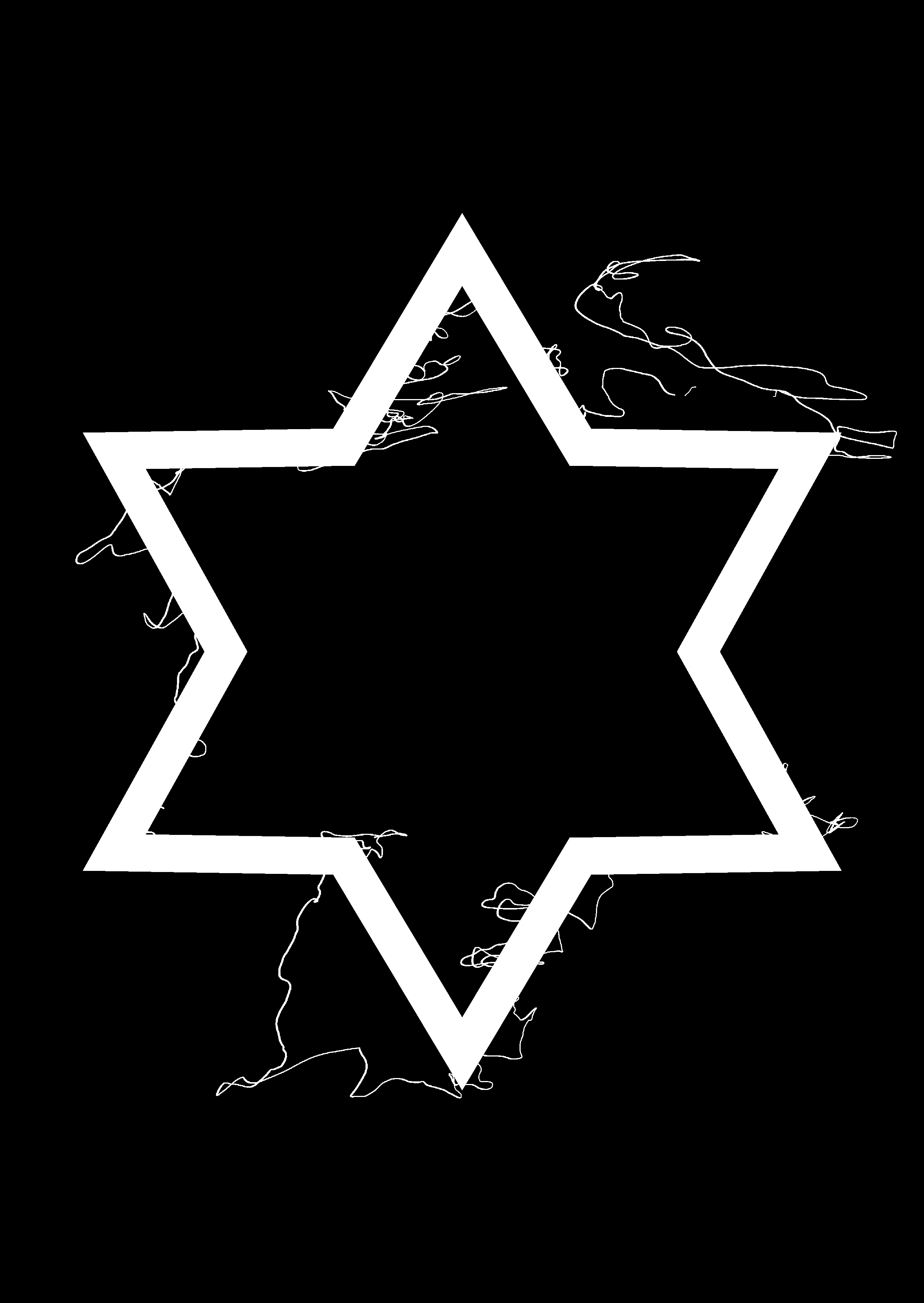

Supplement: Supplementary file 1 [file Data_Sheet_1.ZIP › AnalyzeStarDraw_PythonScript/AnalyzedImages/example_draw_out.png]

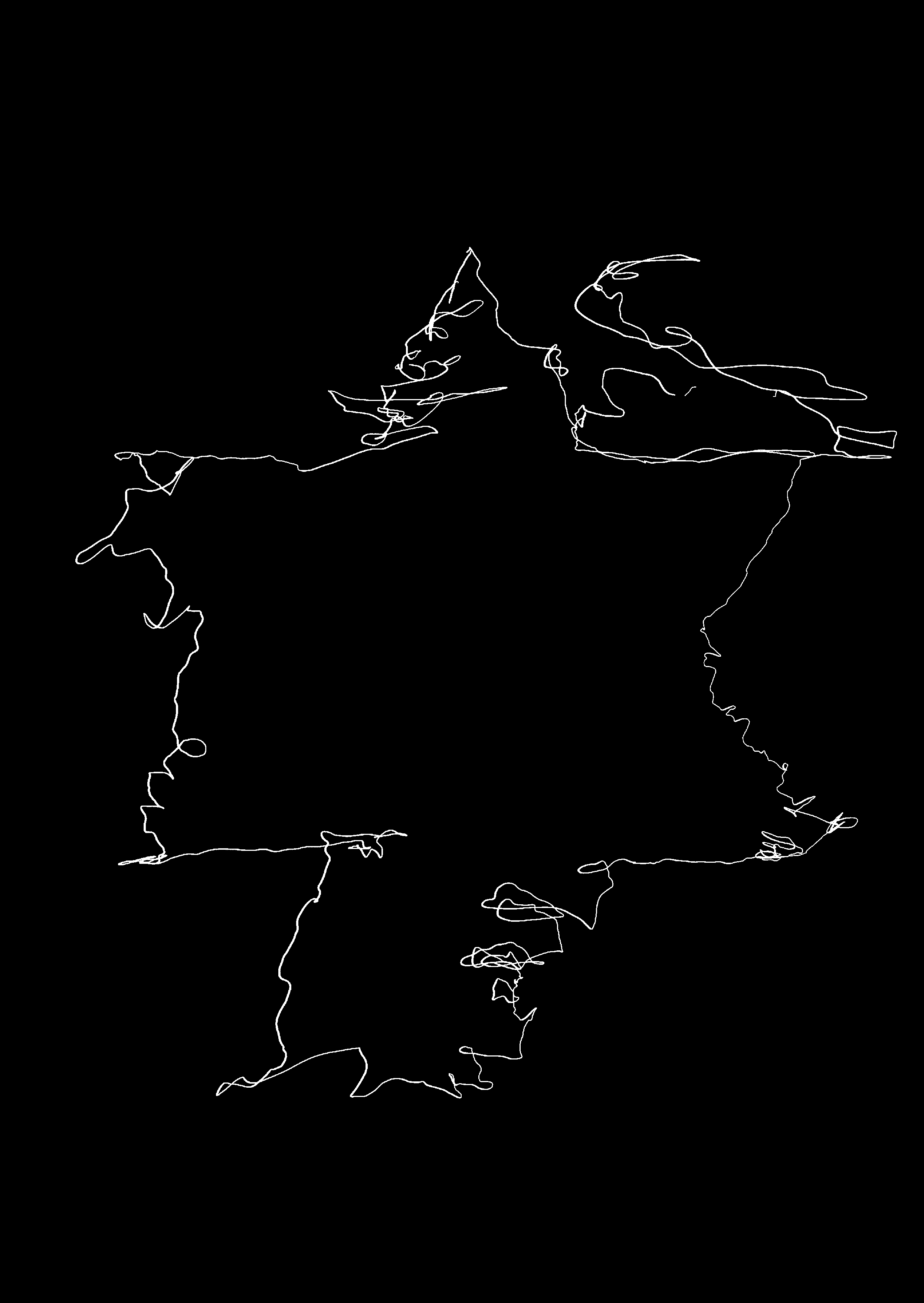

Supplement: Supplementary file 1 [file Data_Sheet_1.ZIP › AnalyzeStarDraw_PythonScript/AnalyzedImages/example_draw__total.png]

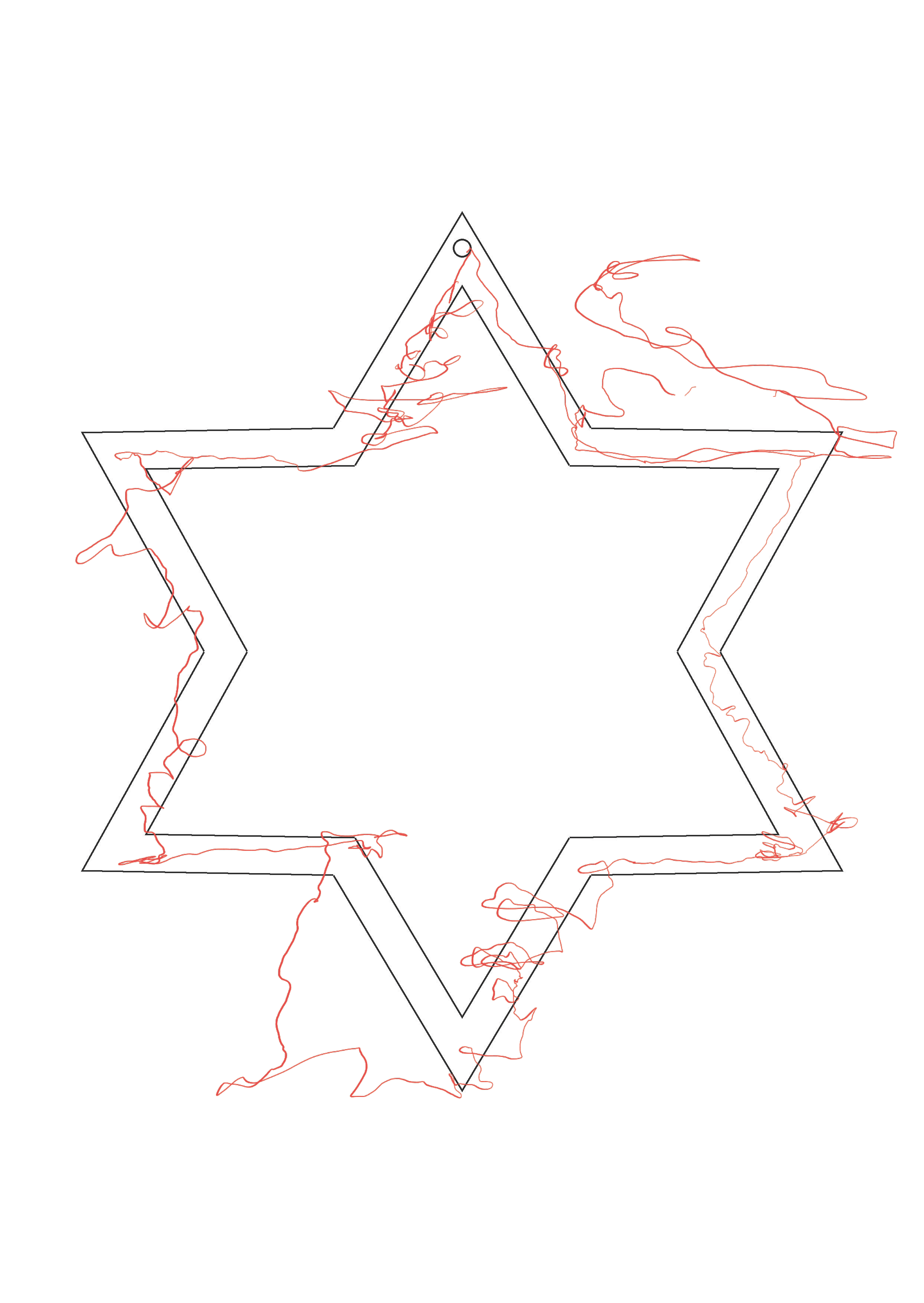

Supplement: Supplementary file 1 [file Data_Sheet_1.ZIP › AnalyzeStarDraw_PythonScript/example_draw.png]

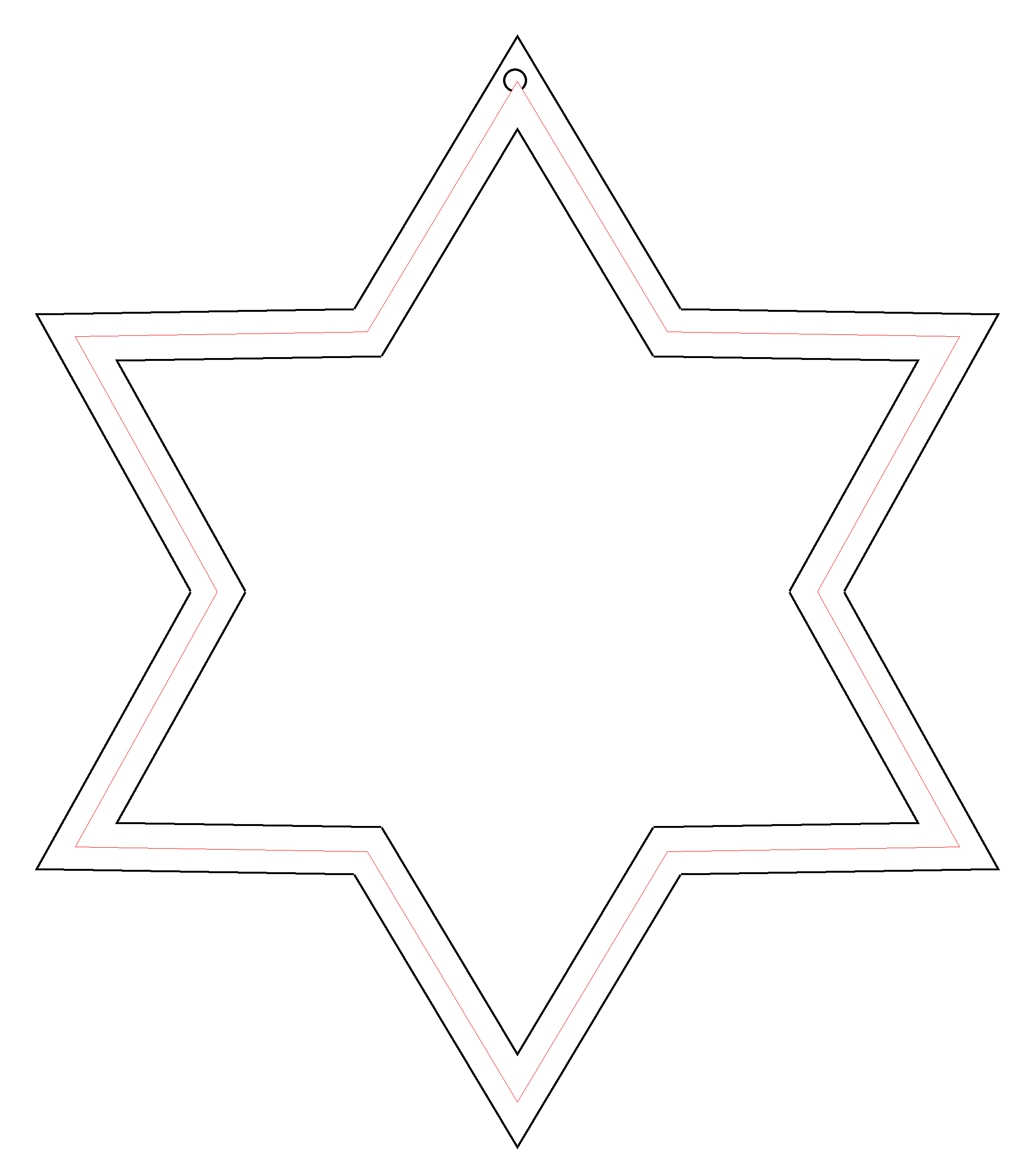

Supplement: Supplementary file 1 [file Data_Sheet_1.ZIP › AnalyzeStarDraw_PythonScript/star_ideal_draw.png]

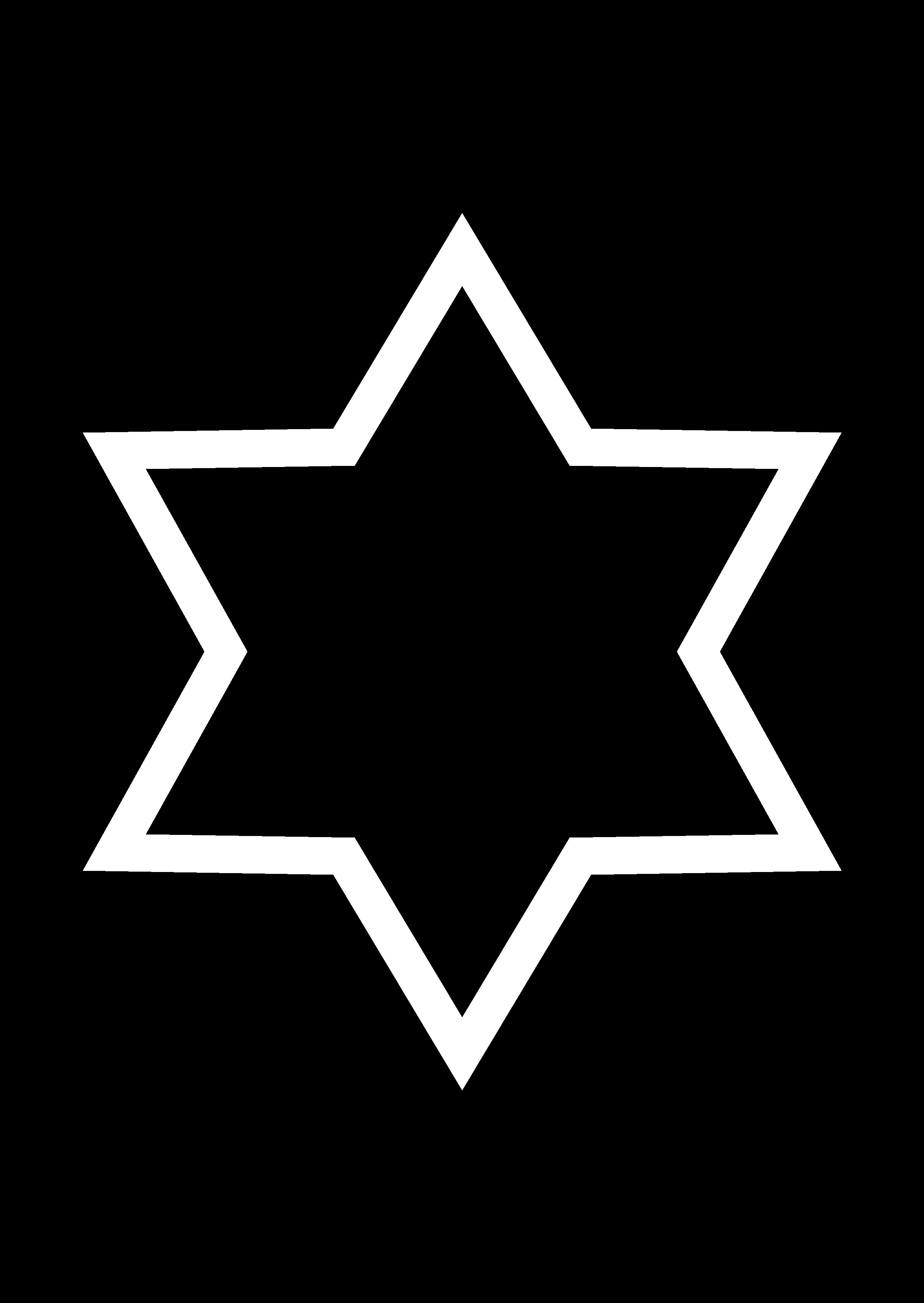

Supplement: Supplementary file 1 [file Data_Sheet_1.ZIP › AnalyzeStarDraw_PythonScript/star_mask.png]

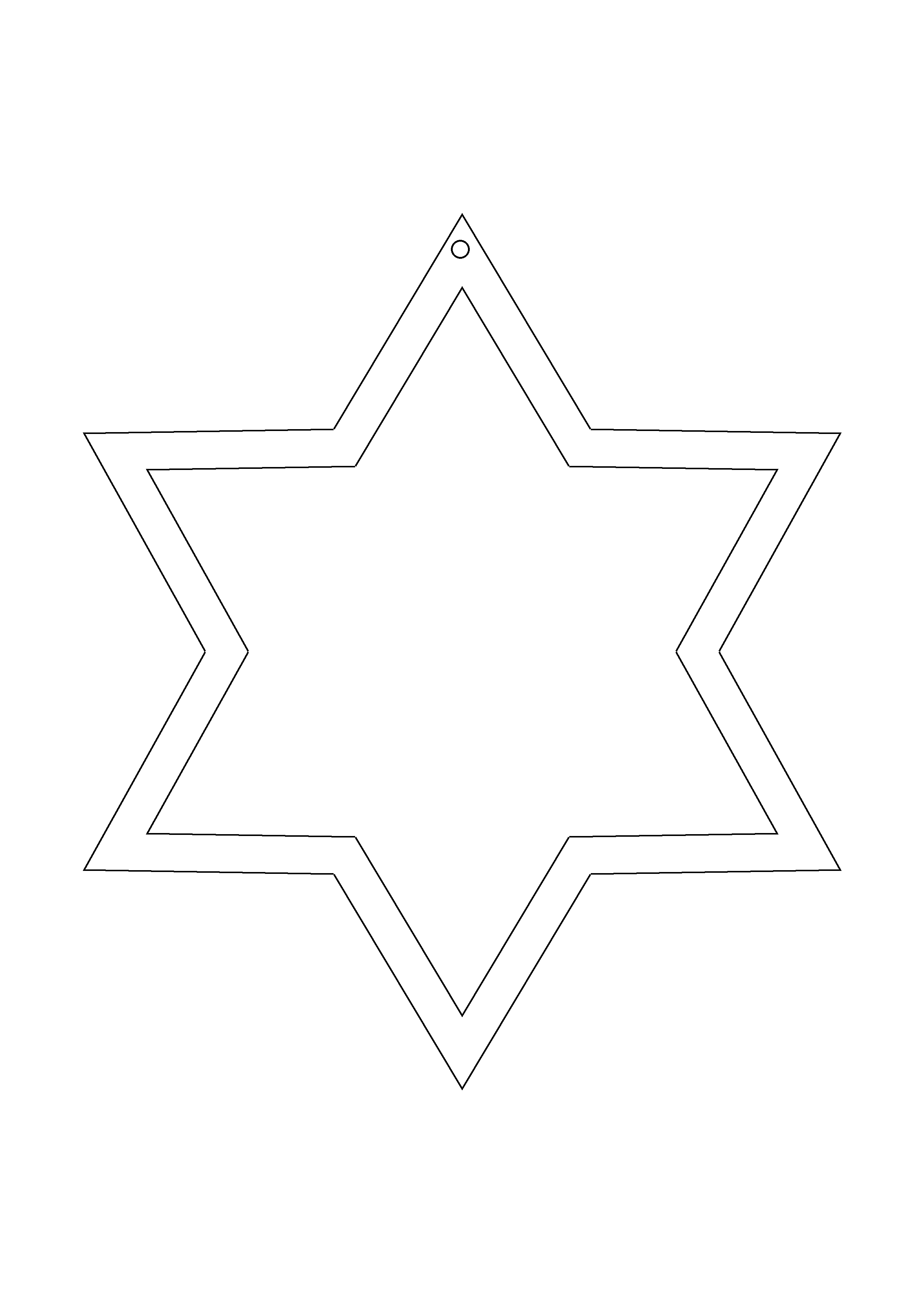

Supplement: Supplementary file 1 [file Data_Sheet_1.ZIP › AnalyzeStarDraw_PythonScript/star_template.png]

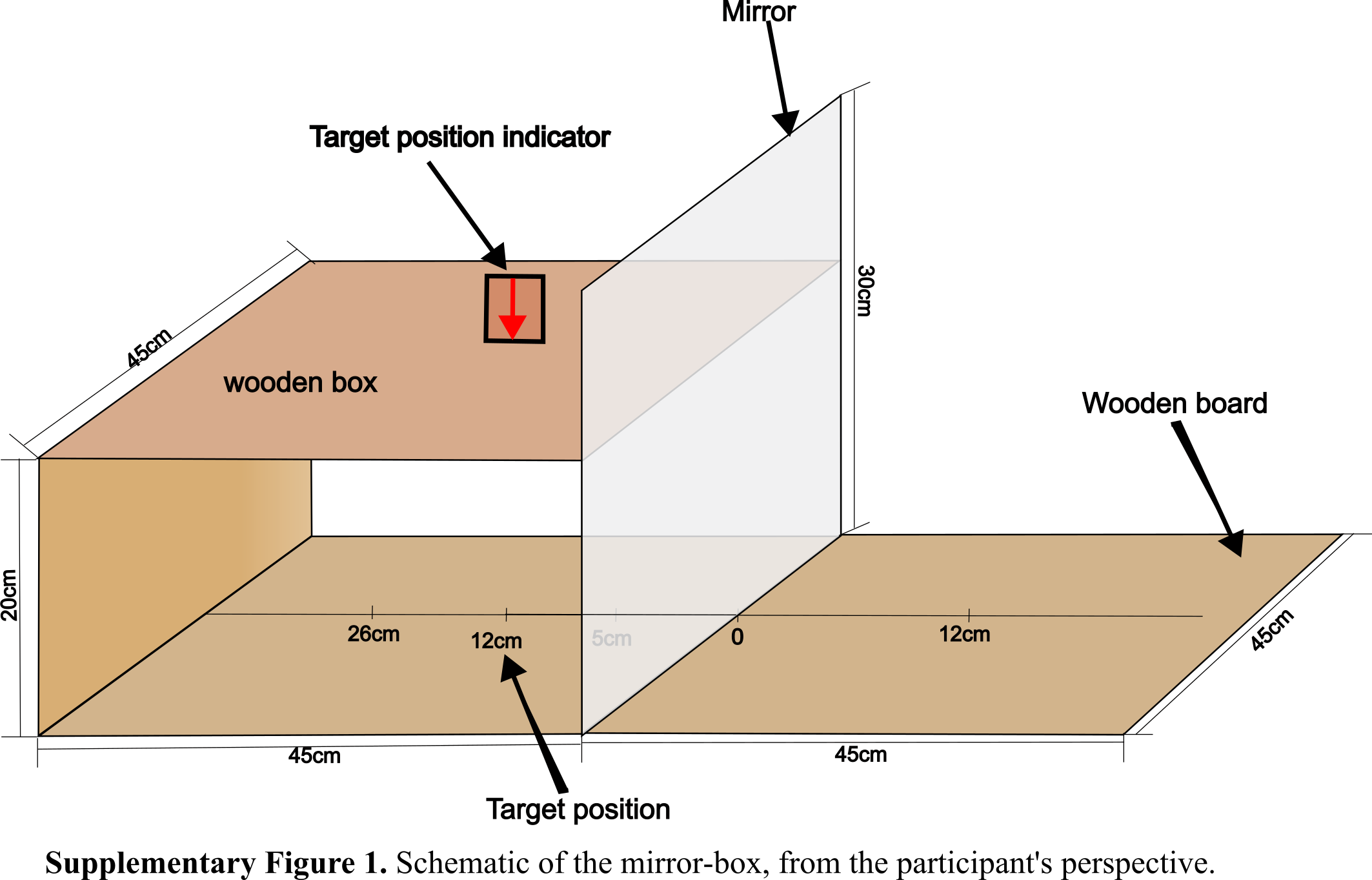

Supplement: Supplementary file 4 [file Image_1.tiff]

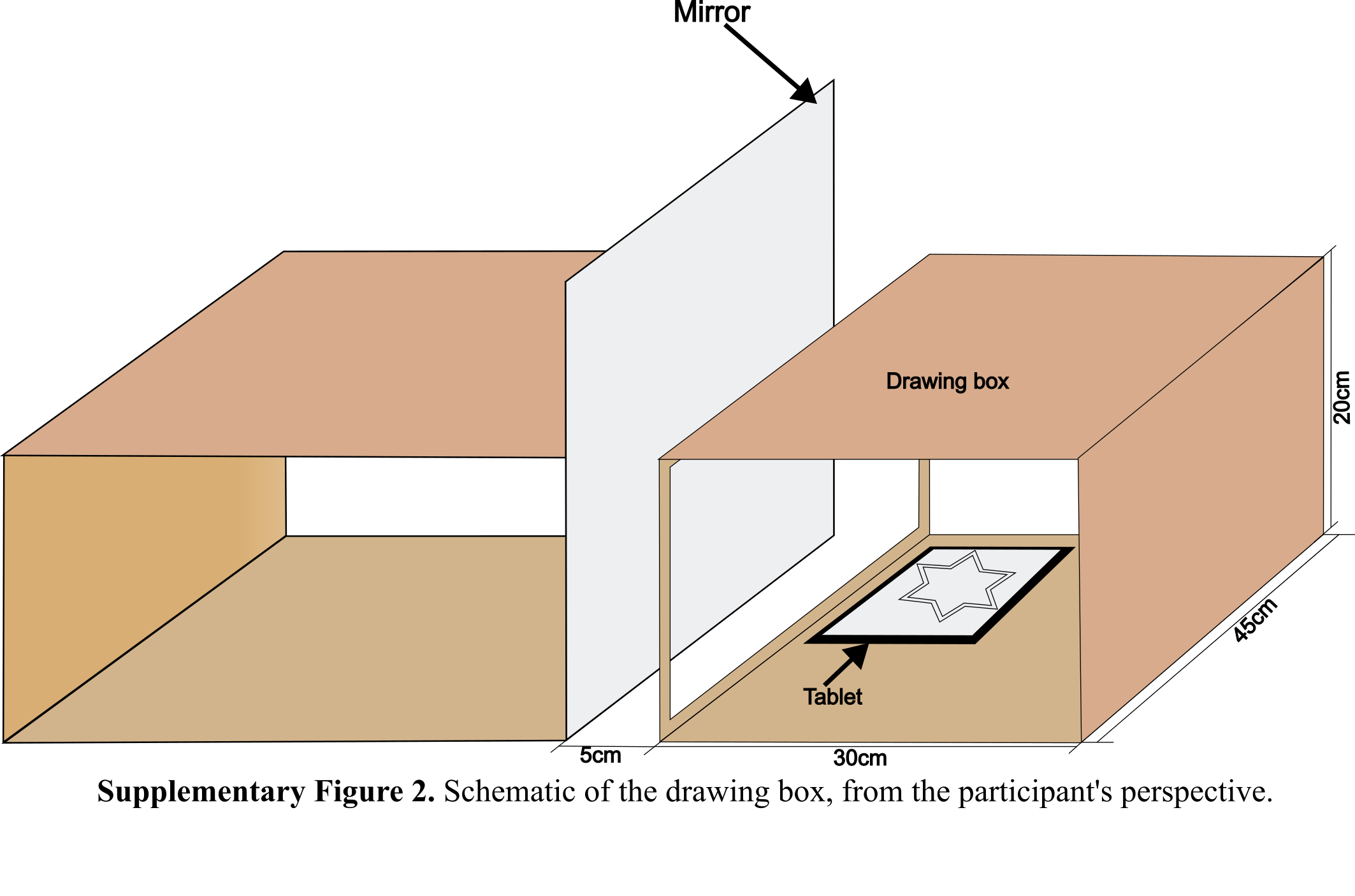

Supplement: Supplementary file 5 [file Image_2.tiff]

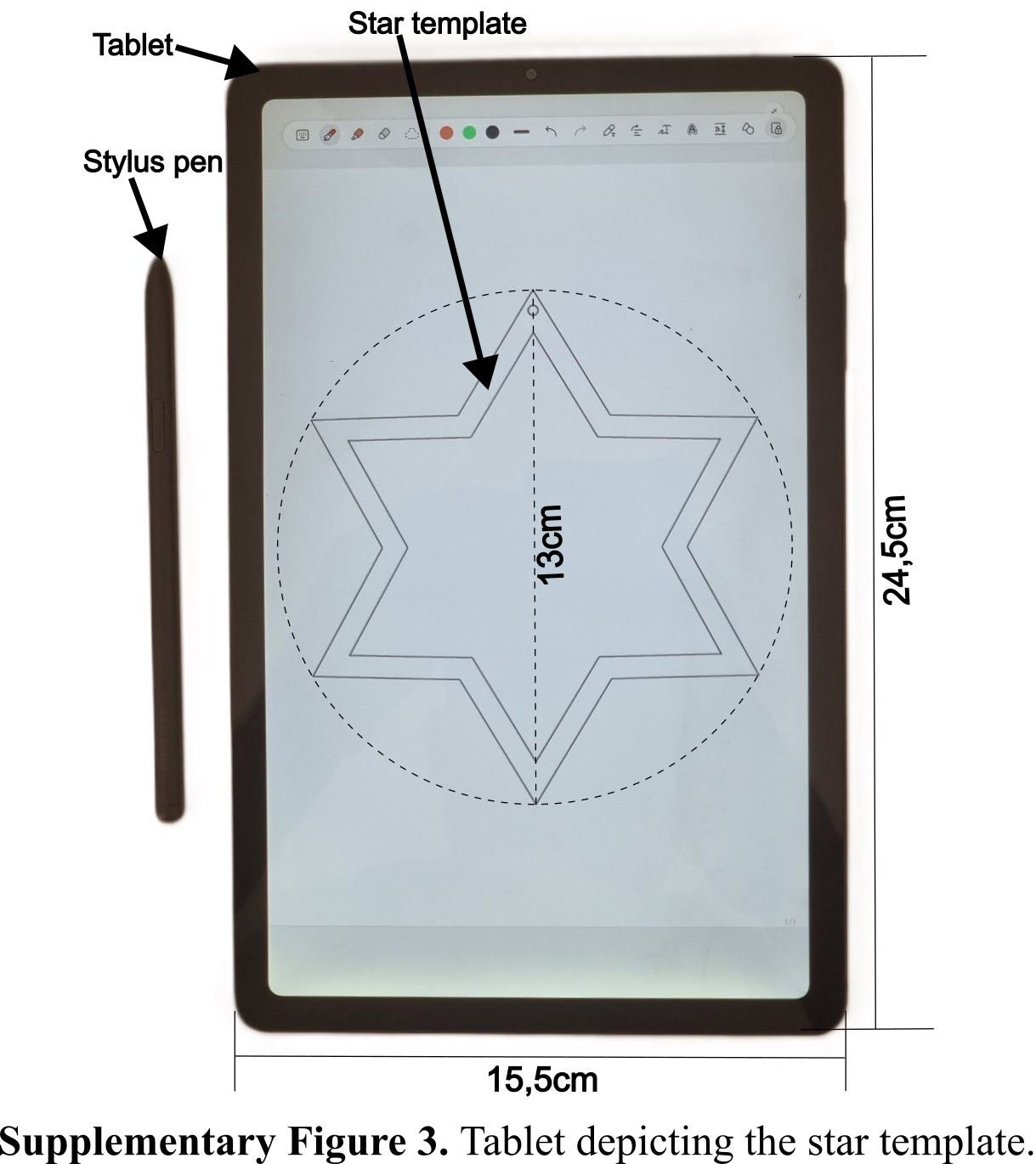

Supplement: Supplementary file 6 [file Image_3.tiff]

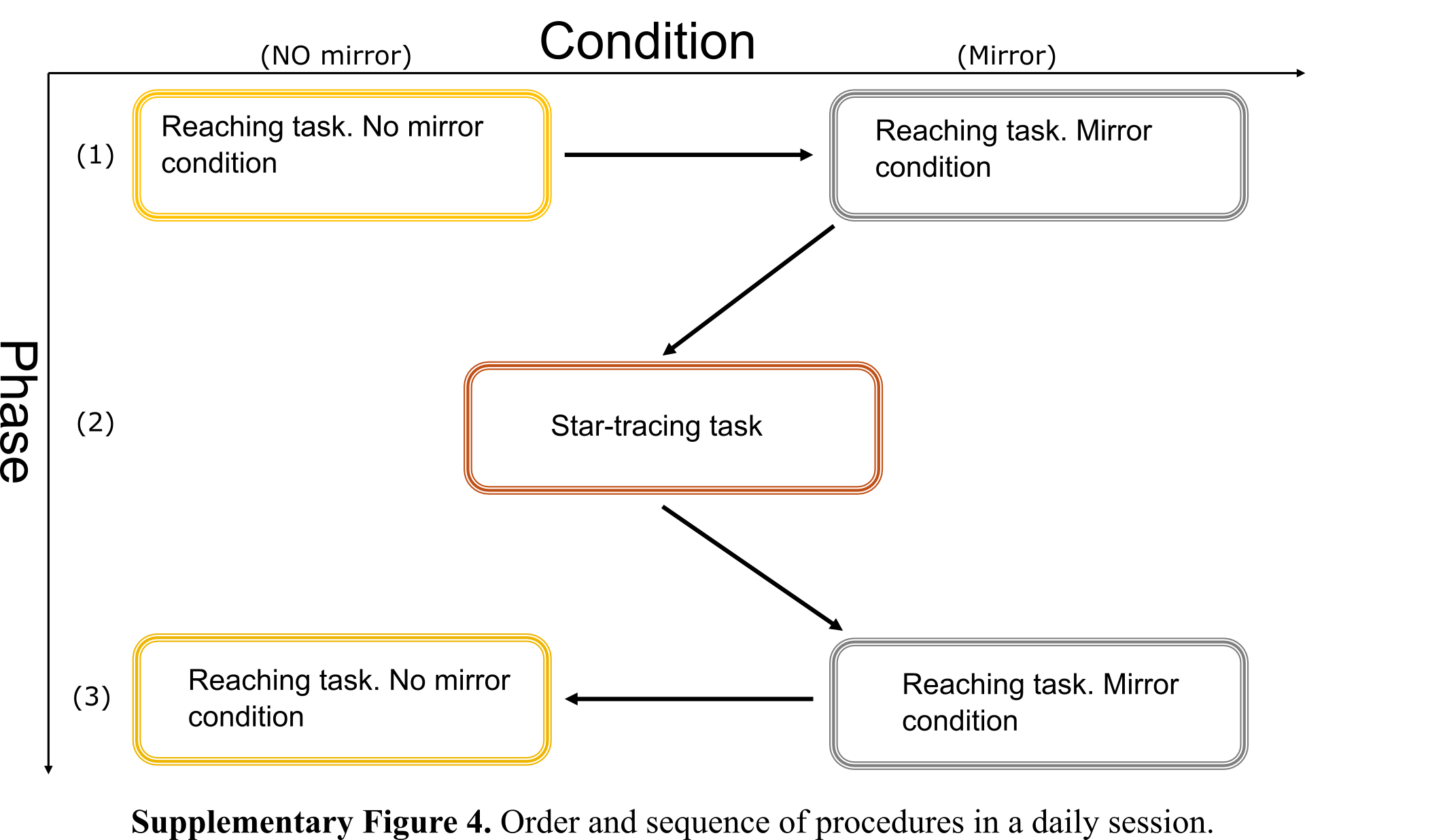

Supplement: Supplementary file 7 [file Image_4.tiff]

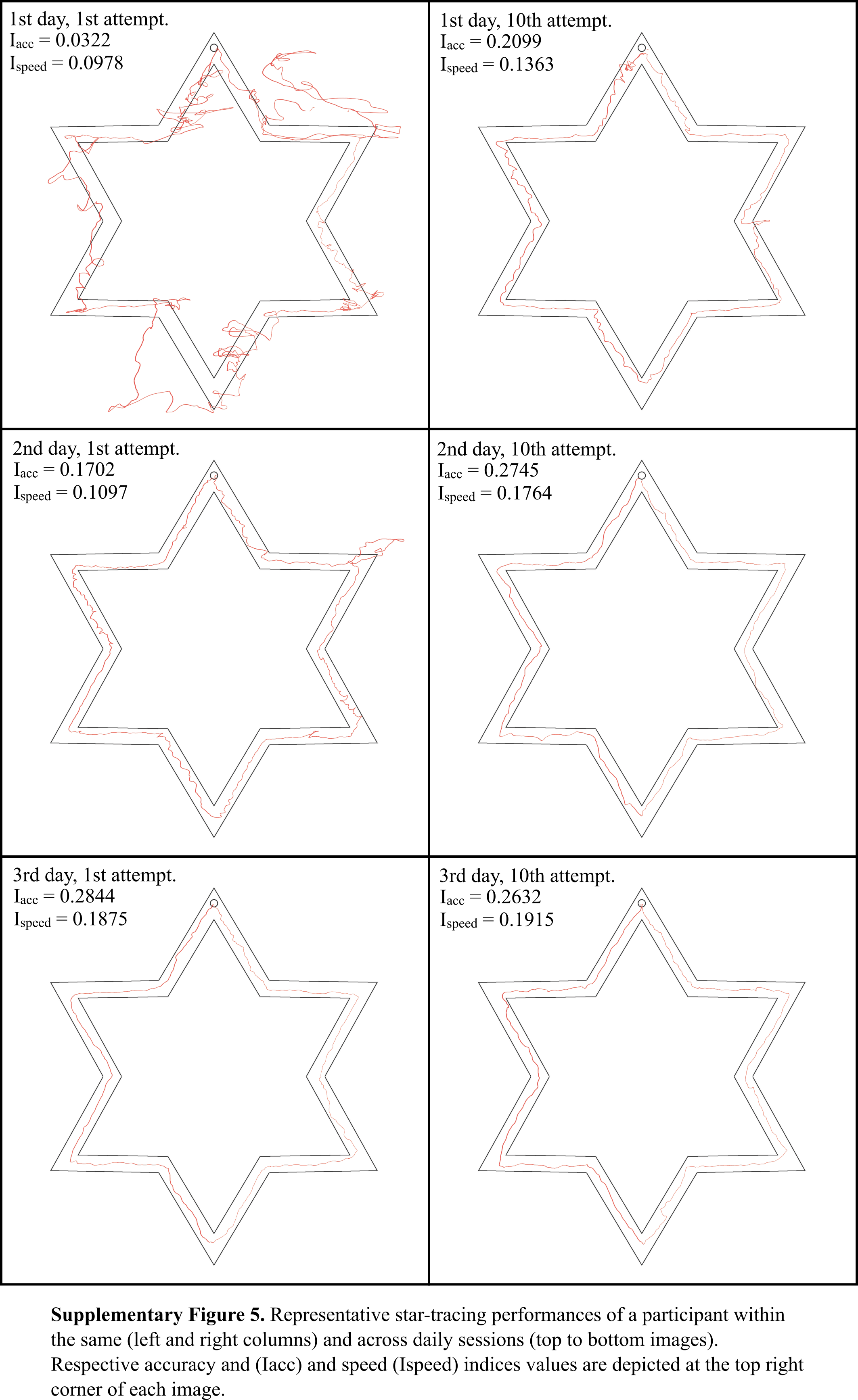

Supplement: Supplementary file 8 [file Image_5.tiff]
